# A causal model of leader ethical behavior and radical innovation: the mediating effect of leader identification and the moderating effect of promotion focus

**DOI:** 10.3389/fpsyg.2025.1467589

**Published:** 2025-04-11

**Authors:** Lingfeng Zhu, Le Wang, Xiu Jin

**Affiliations:** Department of Business Administration, Gachon University, Seongnam, Republic of Korea

**Keywords:** leader ethical behavior, leader identification, promotion focus, radical innovation, relational identification theory, social exchange theory

## Abstract

**Introduction:**

Innovation is essential for an organization's survival and growth, with radical innovation driving transformative change. Despite its significance, the mechanisms that foster employees' radical innovation remain underexplored. This study examines how leader ethical behavior influences radical innovation, offering a novel perspective on leadership's role in fostering innovation.

**Methods:**

A quantitative approach was employed, utilizing survey data from 371 employees of Chinese small and medium-sized enterprises (SMEs). Structural equation modeling (SEM) was applied to test the research model, which links leader ethical behavior to radical innovation, emphasizing the mediating role of leader identification and the moderating role of promotion focus.

**Results:**

The findings reveal that leader ethical behavior positively influences both leader identification and radical innovation. Furthermore, leader identification mediates the relationship between leader ethical behavior and radical innovation. Promotion focus strengthens the effects of leader ethical behavior on radical innovation, as well as the impact of leader identification on radical innovation.

**Discussion:**

This study contributes to radical innovation research by integrating leader ethical behavior into the innovation discourse, extending theoretical perspectives beyond conventional drivers. The findings provide practical insights for organizations seeking to cultivate innovative work environments, enhance leader identification, and empower employees for radical innovation.

## 1 Introduction

The challenges organizations face due to global innovation have become more severe and complex (Jiang et al., 2023). As an enterprise's most important human and intellectual capital, employees' innovative awareness and behaviors are key for enabling organizational development (Wang et al., 2023). To stand out among their fierce competition, companies need their employees to constantly conceive of new and groundbreaking ideas (Liu et al., 2022). Radical innovations cause dramatic shifts in products, services, and processes; these shifts dramatically change the existing market and industry landscape and even give rise to new markets and industries (Miller et al., 2006). Domínguez Escrig et al. (2016) emphasize that organizations can circumvent rigidity by using radical innovation to enter entirely new areas and experiment with new processes. This explains the need for innovation among organization members and the importance of employee radical innovation for organizational survival and growth, reflecting the positive impact employee radical innovation has on an organization.

This study examines and validates a research model that leads to employee radical innovation by emphasizing its importance. Radical innovation entails acquiring and applying new knowledge to develop entirely new products or services for new customers or emerging markets (Benner and Tushman, 2003). It is characterized by the fact that it is set within change and therefore has high levels of high risk and uncertainty. While previous studies have explored variables that can be used to examine innovation, this study emphasizes the role of leader ethical behavior, which is expected to improve employee radical innovation. Leader ethical behavior refers to appropriate behaviors that are demonstrated by leaders through their personal conduct and interpersonal relationships (Brown et al., 2005). Specific examples of these behaviors include honesty, consideration for others, and fair treatment of employees (including respect and voice). Consequently, leader ethical behavior often manifests as altruism. When leaders demonstrate ethical behavior, they not only promote harmonious organizational member relationships, but also motivate team members to exchange knowledge and debate ideas, thus creating the appropriate conditions for radical innovation to occur (Qiao and Qiao, 2023). Moreover, leader ethical behavior allows employees to maintain positive emotions and initiate beneficial interactions with others (Xiu and Zhao, 2016). When employees experience positive emotions, such as happiness, love, and respect, they are motivated to discard time-tested or automatic (everyday) behavioral scripts in favor of novel, creative, and often unscripted paths of thought and action (Fredrickson, 1998). Therefore, leader ethical behavior that puts employees' needs and wellbeing first, making them feel respected and accepted, will strengthen employee-leader relationships and create a positive organizational climate. The resulting positive impact will set the stage for radical innovation.

This study's purpose is to verify whether leader ethical behavior influences radical innovation and determine which factors mediate the process between leader ethical behavior and radical innovation. Leader identification refers to a state that exists when an employee incorporates their perceptions of a leader into their self-concept (Kark et al., 2003). Employees with stronger leader identification are more likely to engage in innovative behaviors then employees without strong leader identification because they tend to view the organization's development as their job (Niu et al., 2018). Therefore, employee work behaviors are largely influenced by their leaders. When leaders demonstrate that they are trustworthy, concerned for employees, make fair decisions, and adhere to normative behaviors, they are likely to win employee trust and respect; this trust and respect helps stimulate strong leader identification. Leader identification prompts employees to pay more attention to developing their relationships with leaders to maintain good interactions between the two parties and to work actively and diligently. They will not only complete the tasks within the scope of their stated duties and responsibilities but are also more likely than their peers to engage in the extra-role behaviors their leaders expect, including demonstrating innovative behaviors (Yang and Shang, 2022). These behaviors lead to radical innovation, which suggests that leader ethical behavior motivates employee radical innovation by stimulating leader identification. We argue that leader identification mediates the relationship between leader ethical behavior and radical innovation.

Research also suggests that promotion focus may affect employee radical innovation. Promotion focus exists when an employee's need for growth and advancement motivates them to strive to align themselves with their ideals and increase the gains to be realized in reaching their desired state (Brockner et al., 2004). Because employees with promotion focus tend to be more fixated on positive event outcomes, their disposition toward their work is exploratory; moreover, they are highly attracted to the development and fulfillment inherent in innovation (Wu and Zhang, 2023). Employees with promotion focus view their work as rewarding because they believe that it helps them realize their job and career potential (Wallace and Chen, 2006). Promotion focus also emphasizes change and motivates employees to engage in innovation, acquisition, and risk-taking oriented behaviors (Xiao and Xu, 2023). Therefore, exploring the moderating role of promotion focus on the relationship between leader ethical behavior and radical innovation and that between leader identification and radical innovation is essential to determine how the interaction changes the level of radical innovation.

Therefore, this study has three primary purposes. First, prior research on how leader ethical behavior guides employee ethical behavior is abundant (Rabie and Malek, 2020; Al Halbusi et al., 2021). In contrast, how leader ethical behavior influences employee radical innovation is relatively understudied, a situation that calls for additional research on radical innovation. Anderson et al. (2014) also suggest that future research should focus on exploring how leadership variables influence radical innovation. Given its importance, this study aims to determine the relationship between leader ethical behavior and radical innovation by investigating and revealing its role in employee radical innovation and to further show how leader ethical behavior can lead to radical innovation, expanding the field of radical innovation research.

Second, based on relational identification and social exchange theories, this study introduces leader identification as a mediating variable. Specifically, relational identification theory emphasizes the leader-employee role relationship; when employees find this relationship attractive, they incorporate the leader's values, goals, and beliefs into their self-concept (Sluss and Ashforth, 2007). Social exchange theory suggests that willingness to give back and associated behaviors are fostered when individuals receive care, support, and trust from others (Bandura, 1986). This payback behavior is not directed only toward the leader but also toward the organization as a whole, since the leader is often seen as the organization's representative (Rhoades and Eisenberger, 2002). This study suggests that employees with high leader identification not only adopt the leader's goals as their own and actively innovate to improve organizational performance, they also reinforce this identification when perceiving the leader's ethical behavior and actively engage in feedback behavior. Therefore, we expect that leader ethical behavior influences employee radical innovation by motivating and strengthening employees' leader identification. This broadens the scope of research on radical innovation.

Finally, while prior studies have explored the moderating effect of employee promotion focus on employee behavior in terms of leadership style, they have all neglected the effect of leader identification on employee promotion focus (Zeng et al., 2018; He et al., 2023). This study is a novel attempt to determine how this interaction affects radical innovation by not only exploring the interaction between leader ethical behavior and promotion focus, but also by evaluating the interaction between leader identification and promotion focus.

This study's overall focus is to develop a research model that leads to radical innovation and argue for the significance of that research model. In addition, it provides a theoretical basis for related research. Thus, the study contributes to expanding the scope of radical innovation research.

## 2 Theoretical background and hypotheses

### 2.1 Leader ethical behavior and radical innovation

Leader ethical behavior refers to leader behavior that is consistent with ethical principles and values (Engelbrecht et al., 2017). Leader ethical behavior can motivate employees to actively participate in their work and make them more proactive and responsible in their work (Lee, 2016). It can also create an organizational environment that is suitable for innovation and encourages and motivates employees to think creatively (Chen and Hou, 2016). Intuitively, when leaders engage in ethical behavior, employees will be more positive, hopeful, and optimistic about their organization and work environment and will be more willing to stay and contribute to the organization's success (De Hoogh and Den Hartog, 2008). Therefore, this study suggests that leader ethical behavior may be a precondition for employee radical innovation. Radical innovation involves employees developing and implementing new ideas that are completely different from an organization's existing management practices or business processes (Zhang et al., 2014). Leaders' altruistic behaviors can promote radical innovation (Domínguez Escrig et al., 2016).

Employees often face a range of challenges generating and implementing new ideas, and leader ethical behavior plays a critical role in this process by promoting work ethics, autonomy, and self-accountability, which can be considered potential predictors of innovation (Shafique et al., 2020). Moreover, leader ethical behavior generally focuses on employee needs and wellbeing as a primary concern, which helps employees feel respected and accepted; it can also shape a safe organizational climate, providing the basis for radical innovation (Yoshida et al., 2014). Accordingly, leaders who engage in two-way open communication with their employees, listen to them sincerely and patiently, and encourage them to express their concerns and opinions can greatly contribute to the emergence of new ideas to improve existing work processes (Martins and Terblanche, 2003). Therefore, leader ethical behavior makes employees feel cared for, safe, and respected; acceptance of these behaviors is expected to enhance their courage and motivation to innovate. Consequently, employees will be happy to perform positively in their organization and motivated to advance radical innovation. Based on this, we offer the following hypothesis.

Hypothesis 1. Leader ethical behavior will have a positive influence on radical innovation.

### 2.2 Leader ethical behavior and leader identification

Leader identification is a state in which employees define themselves based on their relationship with their leader (Guo et al., 2020). That is, employees see their leaders as role models and develop an attitude of recognition and acceptance toward them (Cui et al., 2021). Moreover, leader identification motivates employees to think proactively from the leader's perspective (Peng et al., 2020). Leader identification becomes more significant when employees and their leaders have similar values (Marstand et al., 2018). Employees are likely to be attracted to their leader's attitudes and behaviors if the leader engages in ethical behavior. Leader ethical behavior leads to positive interactions between employees and their leader; a strong personal attachment to the leader will be created through constant interactions and communication, stimulating high levels of leader identification (Niu and Liu, 2021a). Sluss and Ashforth (2007) research also suggests that employees with high leader identification pay more attention to building good interpersonal relationships with their leaders, increasing their understanding of and immersion in the organization.

Therefore, leader ethical behavior often generates positive perceptions and ethical role modeling among employees, motivating them to identify with their leaders (Lee, 2016). Specifically, leaders support and encourage employees so that they sense respect and recognition, satisfying employees' need for self-esteem. Hence, employees also reinforce respect and trust in their leaders and may develop higher levels of leader identification (Hao and Sun, 2020).

In terms of organizational climate, ethical behavior emphasizes a leader's integrity, selflessness, and accountability to the organization's employees, which creates a trustworthy, supportive, and encouraging atmosphere that is more likely to evoke leader identification among employees (Gu et al., 2015). Simply put, leader ethical behavior demonstrates integrity and altruism, which makes employees feel respected and cared for; at the same time, it inspires and reinforces the leader's personal charisma. This role model effect establishes a positive example in the organization, which in turn inspires employee leader identification. From this summary, we propose the following hypothesis.

Hypothesis 2. Leader ethical behavior will have a positive influence on leader identification.

### 2.3 Leader identification and radical innovation

Employees who identify with their leaders connect closely to those leaders, align their feelings and beliefs toward the leaders, and exhibit appropriate attitudes and behaviors (Cui et al., 2021). Specifically, employees with high levels of leader identification are motivated to respond positively to their work because of their recognition and respect for their leaders (Yoshida et al., 2014). In contrast, employees with low levels of leader identification disapprove of their leaders' behaviors and disrespect or are fed up with their leaders, which can lead to psychological disengagement and negative behaviors (Wang et al., 2021). Peng et al. (2020) find that high levels of employee leader identification stimulate employee creativity because leader identification gives them a sufficient sense of psychological safety; this makes them feel that it is safe to try out new ideas and increases their willingness to engage in high-risk radical innovation. Thus, radical innovation inherently requires that employees have a flexible mindset and willingness to take risks (Wu and Zhang, 2023).

Employees who strongly identify with their leader will see the leader's goals as their own and voluntarily give their all for the leader's benefit and success (Liao et al., 2019), which can trigger radical innovation. Accordingly, the stronger an employee's leader identification, the more inclined they are to build positive relationships with their leaders and work more toward achieving organizational performance goals; at work, they are likely to more actively seek help and guidance, exploring and expanding new ways of doing things, and displaying greater innovative awareness and behaviors (Niu and Liu, 2021b). Even if organizational goals do not fit employee interests, strong identification with their leaders creates keen personal motivation for them to begin working toward innovation (Yoshida et al., 2014).

Overall, leader identification is more likely to result in a close working relationship and greater willingness to share information and resources. Employees will be and are more willing to invest their time and energy in working hard for the organization's success because they identify with the leader's vision and goals. Therefore, this suggests that leader identification is a positive factor in promoting employee radical innovation. Based on this, we suggest the following hypothesis.

Hypothesis 3. Leader identification will have a positive influence on radical innovation.

### 2.4 The mediating effect of leader identification

Leader identification is based on a deep understanding of and respect for their leader. Leader identification also influences employees to initiate positive behaviors that promote organizational development (Marstand et al., 2021). Specifically, employee leader identification can be fused into organizational identification through cognitive, affective, and behavioral processes, and this fusion mechanism can be influenced by leader characteristics (Sluss et al., 2012). Because leader ethical behavior demonstrates strong dedication and active defense of collective interests, employees who identify strongly with their leaders may view them as team or organization representatives (Gu et al., 2015). As a tangible manifestation of their sense of belonging to the organization and through this sense of belonging, employees who identify with their organization are likely to be motivated to exhibit innovation (Tang and Naumann, 2016).

Leader ethical behavior also contributes to a positive organizational climate in which employees are more willing to take responsibility and proactively report problems in the organization (Brown et al., 2005). Ethical behavior, which emphasizes treating employees fairly and focusing on meeting their needs, creates a positive perception among employees that leaders are genuinely concerned about their wellbeing; this, in turn, inspires leader identification (Svendsen et al., 2020). After employees develop leader identification, they tend to translate the self-concept of leader identification into actual attitudes and specific behaviors, which may be used as feedback through demonstrations of higher levels of creative work (Hao and Sun, 2020). Related to this, leaders displaying leader ethical behavior makes employees feel good about their leaders, which strengthens the respect and trust for their leaders, allowing employees to easily identify with their leaders (Peng, 2020). Leader identification reflects the existence of a high-quality relationship between an employee and a leader, increasing the employee's sense of safety; subsequently, employees feel that innovation is less risky, making them more willing to attempt to execute their innovative ideas (Gu et al., 2016).

Therefore, in this study, we argue that when employees experience leader ethical behavior, they feel care, trust, and justice, which leads them to agree with their leader's ideas and expectations and form a sense of identification with the leader. This not only reduces an employee's perceived risk of innovation but also stimulates their desire for rewards and increases radical innovation behavior. From this summary, we propose the following hypothesis.

Hypothesis 4: Leader identification will mediate the relationship between leader ethical behavior and radical innovation.

### 2.5 The moderated effects of promotion focus

Promotion focus exists when employees are aligned with their self-ideals, view risks positively, and pursue positive outcomes (Higgins, 1997). Employees with promotion focus are eager to achieve and gain, specifically wanting to realize “success” and actively avoiding negligence or mistakes (i.e., loss of accomplishment) (Higgins, 1997). Employees with high promotion focus view difficulties at work as challenges and act quickly to deal with them positively and through their own efforts (Liao et al., 2022). Simply put, employees with promotion focus are concerned with development and growth and are more inclined to initiate change to achieve their goals.

Leadership behavior is an important signal in an organization and draws employees' attention to promotion focus (Brockner et al., 2004). Leader ethical behavior is admired, respected, and trusted by employees, motivating them to develop promotion focus, have a more positive attitude toward their work, and pursue high levels of work outcomes (Lai et al., 2018). In Tung's (2016) study, the higher the promotion focus, the greater the capacity for employee creativity. Promotion focus increases employees' hunger for success; they thus value the opportunities in their organizations that may lead to success, making them more likely to innovate to achieve personal and organizational development (Xiao and Xu, 2023). We therefore suggest that employee promotion focus will play a moderating role in the relationship between leader ethical behavior and employee radical innovation. Specifically, employees with promotion focus who feel cared for and respected by their leaders are more likely to focus on pursuing opportunities for growth, gain, or success than on maintaining the status quo. Moreover, for employees with strong promotion focus, high demands lead to better outcomes in creative generation and insight tasks to promote radical innovation (Sacramento et al., 2013). Overall, in the presence of leader ethical behavior, promotion focus employees are more willing to take risks to express and initiate shifts in or innovations to the status quo. In contrast, promotion focus employees will be unable to take risks when they perceive rude and abusive leader behaviors and will not engage in challenging actions to achieve their goals (Kim et al., 2020). Therefore, we propose that strong employee promotion focus strengthens the relationship between leader ethical behavior and employee radical innovation.

Leader identification affects organization members' emotional experiences and behavioral motivation, which influences their subsequent behavior (Liao et al., 2019). Radical innovation means developing and applying entirely new technologies and processes in an organization (Moors and Vergragt, 2002), accompanied by higher risk and uncertainty. However, when employees adopt promotion focus, they tend to be willing to take greater risks in pursuing their goals (Wallace and Chen, 2006). When employees have promotion focus, the more they identify with their leaders, the higher their emotional safety. This emotional safety is reflected in more prominent self-esteem, a sense of belonging, and openness, which makes employees more comfortable expressing their views on issues. Employees will be more focused on realizing their personal ideals and passions through their work, actively looking for growth and advancement and striving to achieve radical innovation (Ryou and Kim, 2018). When employees with promotion focus develop leader identification, they use the leader as a role model, which motivates them to achieve the same. They focus more on pursuing success; by imitating and learning from their leader, employees will display exploratory tendencies during the work process and be willing to try new tasks and problem solving approaches, increasing the innovation level (Li and Shang, 2011). Therefore, this study suggests that employee promotion focus strengthens the relationship between leader identification and employee radical innovation.

Overall, through the optimism and sense of innovation brought about by promotion focus, not only will employees be able to eliminate concerns and participate more actively in innovation when their leaders demonstrate ethical behavior, but this sense of innovation will be further strengthened through leader identification. Therefore, we propose that promotion focus not only plays a positive role in moderating the relationship between leader ethical behavior and radical innovation but also has a positive role in moderating the relationship between leader identification and radical innovation.

Hypothesis 5: Promotion focus will have a positive moderating effect on the relationship between leader ethical behavior and radical innovation.
Hypothesis 6: Promotion focus will have a positive moderating effect on the relationship between leader identification and radical innovation.

## 3 Methods

### 3.1 Sample characteristics

This study focused on organizational members who work in Chinese SMEs and conducts a survey through an online questionnaire. The invitation to the survey clearly stated the purpose of the study and emphasized the anonymous and voluntary nature of the survey, while promising that there would be no adverse effects on the individual respondents and the organizations they belong to. In particular, none of the participants in the survey were minors, and the survey was conducted after obtaining prior consent from all participants. In addition, the questionnaire consists of five sections, with core content covering leader ethical behavior, leader identification, promotion focus, radical innovation, and demographic information. To ensure that Chinese SME employees are able to respond successfully, all survey questions have been accurately translated and rationalized to fit the local language and cultural context. Specifically, in order to ensure the accuracy of the content of the measurement tool, the English version was first translated into Chinese. Then, the Chinese was translated back into English to confirm the accuracy of the content once again. Therefore, the survey was conducted in a version translated into Chinese. A total of 371 data samples were collected and used for empirical analysis. Regarding the demographic characteristics of this study, there were 148 (39.9%) males and 223 (60.1%) females.

Regarding age, 1 (0.3%) people were under 20 years old, 63 (17.0%) were 20 to 29 years old, 80 (21.5%) people were 30 to 39 years old, 123 (33.2%) people were 40 to 49 years old, and 104 (28.0%) people were 50 or over.

Regarding education, 122 (32.9%) people had finished technical secondary schools or high schools, 78 (21.0%) were junior college graduates, 89 (24.0%) people were college graduates, 17 (4.6%) people had master's degrees, 3 (0.8%) person was a doctor, and 62 (16.7%) were other.

In terms of employment relationships, full-time jobs were the most numerous at 230 (62.0%) and informal positions were 141 (38.0%).

Regarding Service Years, 30 (8.0%) people had worked for a year or under, 47 (12.7%) had worked for 1 to 3 years, 47 (12.7%) had worked for 3 to 5 years, 40 (10.8%) had worked for 5 to 7 years, and 207 (55.8%) people had worked for 7 or over.

Regarding about the time to work with the current immediate leader, 53 (14.3%) people had worked for a year or under, 43 (11.6%) had worked for 1 to 2 years under, 58 (15.6%) had worked with the current immediate leader for 2 to 3 years under, 33 (8.9%) had worked with the current immediate leader for 3 to 4 years under, 36 (9.7%) worked with the current immediate leader for 4 to 5 years under, and 148 (39.9%) people had worked with the current immediate leader for 5 or over.

Regarding enterprise type, 30 (8.0%) people were working in education, 41 (11.1%) people were working in finance, 25 (6.7%) people were working in medical industry, 80 (21.6%) people were working in catering services, 41 (11.1%) people were working in coal mining, 9 (2.4%) people were work in media and 145 (39.1%) people were working in other occupations.

### 3.2 Measurement

Leader ethical behavior is when a leader engages in appropriate behavior that is ethical (Demirtas, 2015). To measure leader ethical behavior, this study used a tool from Lin et al. (2016). The measurement tool consists of 8 items. Sample items include: “My leader looks out for the best interests of his or her employees” and “My leader sets an example for employees in terms of ethics.”

Leader identification is an individual identity, a state in which employees define themselves based on the identity and behavioral performance of their leaders (Cui et al., 2021). To measure the employees' leader identification in small and medium-sized enterprises (SMEs) in China, this study used the measurement scale in Shamir et al. (1998). The measurement tool consists of 7 items. Sample items include: “I respect my leader” and “My values are similar to those of my leader.”

Promotion focus means that employees who have aspirations or hopes as goals and focus on the emergence of positive outcomes will naturally move forward to achieve their goals (Tung, 2016). To measure the promotion focus of employees in SMEs in China, this study used the tool in Wallace and Chen (2006), The measurement tool consists of 6 items. Sample items include: “I can accomplish a lot at work” and “I can also do my job well in a short period of time.”

Radical innovation refers to new, disruptive ideas from employees that are different from the organization's existing framework or processes and that create significant differences from the organization's existing products, services, business processes, and management practices (Madjar et al., 2011). To measure the radical innovation of employees in SMEs in China, this study used the tool in Li et al. (2008). The measurement tool consists of 4 items. Sample items include: “I often create brand new business solutions” and “I am a creator of new techniques and technologies.”

**Figure 1:**
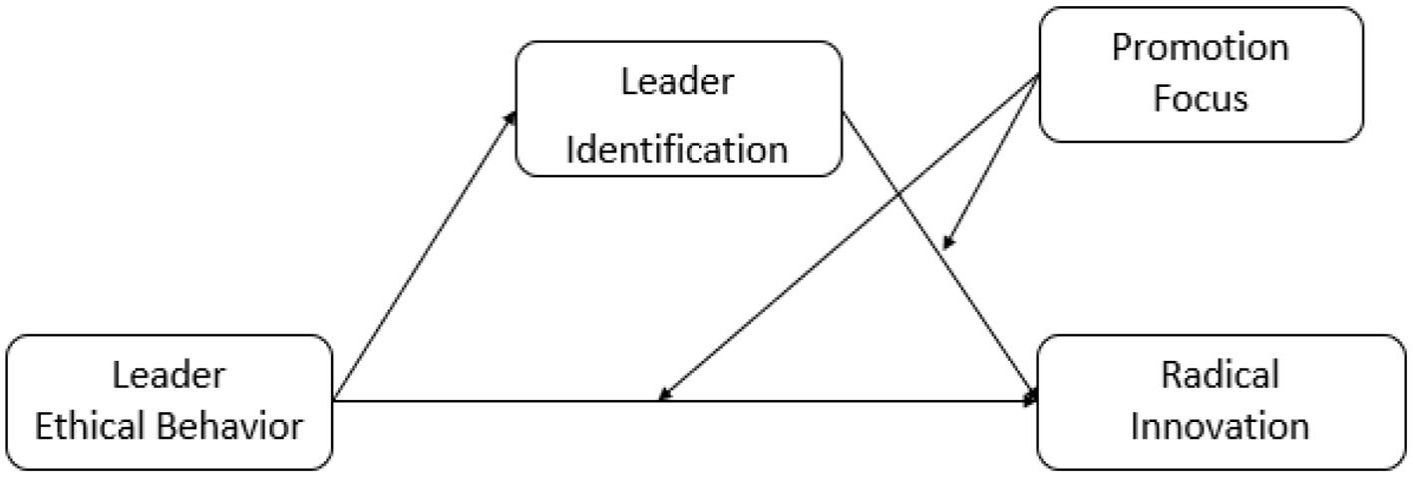
Research model 1.

All items were measured on a seven-point Likert scale, with responses ranging from “1 (strongly disagree)” to “7 (strongly agree).” The higher the score, the stronger the intent. The research model is shown in Figure 1.

## 4 Results

### 4.1 Confirmatory factor analysis

The confirmatory factor analysis (CFA) of Model 1 (four-factor model) showed that the scale was a good fit and had adequate construct validity. Next, we tested convergent validity. The results were as follows: The standardized regression weights of leader ethical behavior ranged from 0.792 to 0.928, leader identification from 0.826 to 0.928, radical innovation from 0.857 to 0.96, and promotion focus from 0.846 to 0.90. Furthermore, average variance extracted (AVE) reflects the proportion of variance that a latent variable captures from its observed variables. The average variance extracted (AVE) for leader ethical behavior was 0.801, leader identification was 0.782, radical innovation was 0.824, and promotion focus was 0.762. These values were all >0.5. Construct reliability (CR) evaluates how well multiple observed variables collectively represent a single latent variable, indicating the overall internal consistency of the scale. The value of the composite reliability (CR) of leader ethical behavior was 0.960, leader identification was 0.950, radical innovation was 0.919, and promotion focus was 0.940. All these values were >0.7. A measurement is considered to have significant validity if the AVE of variables is higher than 0.5 and CR is higher than 0.7 (Jin and Hahm, 2021).

Furthermore, we examined three types of model fit indices: the absolute fit index, incremental fit index, and parsimonious adjusted index. First, the absolute fit index was *X*^2^ (*p*) = 1108.36 (0.000), *X*^2^/df = 4.33, and RMSEA = 0.095. The RMSEA is indeed a “badness of fit” index, with values very close to 0 indicating almost perfect fit and greater values indicating worse fit. For the RMSEA, values <0.05 reflect a small approximation error, values between 0.05 and 0.08 reflect an acceptable error of approximation, and those >0.10 constitute poor model fit (Browne and Cudeck, 1992). Second, the incremental fit index was IFI = 0.943 and CFI = 0.943. Third, the parsimonious adjusted index was PNFI = 0.791 and PGFI = 0.640. Based on these results, the CFA indicates that the measures satisfy the requirements for acceptability (Jin and Hahm, 2021). Therefore, the structural equation model was found to be significant. Table 1 provides the results of the convergent validity tests. For the reliability analysis, when the Cronbach's Alpha coefficient base is above 0.7, reliability is guaranteed. The results of the reliability analysis of each variable in this study are as follows: leader ethical behavior (0.978), leader identification (0.974), radical innovation (0.960), and promotion focus (0.962). All values are above 0.7, thus, verifying confidence in each variable. Table 1 shows the results.

**Table 1:** The result of confirmatory factor analysis.

**Variables**		**Effect**	**SE**	**CR**	***p***	**Standardized regression weights**	**AVE**	**CR**	**Cronbach's alpha**
Leader ethical behavior	A1	1				0.887	0.801	0.960	0.978
	A2	0.903	0.038	23.742	^***^	0.792			
	A3	1.092	0.031	35.313	^***^	0.928			
	A4	1.079	0.032	33.936	^***^	0.902			
	A5	1.097	0.032	34.414	^***^	0.921			
	A6	1.083	0.032	33.547	^***^	0.915			
	A7	1.015	0.032	31.492	^***^	0.901			
	A8	0.998	0.031	32.309	^***^	0.907			
Leader identification	B1	1				0.926	0.782	0.950	0.974
	B2	0.905	0.028	32.784	^***^	0.855			
	B3	1.048	0.028	37.826	^***^	0.935			
	B4	1.072	0.025	42.585	^***^	0.958			
	B5	0.95	0.038	24.88	^***^	0.826			
	B6	0.986	0.039	25.16	^***^	0.827			
	B7	1.011	0.037	27.541	^***^	0.855			
Radical innovation	C1	1				0.857	0.824	0.919	0.960
	C2	1.092	0.031	34.753	^***^	0.925			
	C3	1.266	0.041	30.782	^***^	0.96			
	C4	1.298	0.048	27.099	^***^	0.887			
Promotion focus	D1	1				0.863	0.762	0.940	0.962
	D2	0.913	0.035	25.78	^***^	0.859			
	D3	0.909	0.033	27.239	^***^	0.878			
	D4	1.004	0.04	24.868	^***^	0.846			
	D5	0.947	0.033	28.435	^***^	0.891			
	D6	0.964	0.036	26.438	^***^	0.900			
Model fit index		*X^2^*(*p*) = 1108.36 (0.000), *X^2^*/df = 4.33, RMSEA = 0.095, IFI = 0.943, CFI = 0.943, PGFI = 0.640, PNFI =0.791							

*** *p* < 0.001 indicates that all factor loadings are statistically significant.

### 4.2 Descriptive statistics and correlation analysis

The descriptive statistical analyses included means and standard deviations. The mean values of leader ethical behavior, leader identification, radical innovation, and promotion focus were 5.645, 5.703, 5.293, and 5.698, respectively. The standard deviations of leader ethical behavior, leader identification, radical innovation, and promotion focus were 1.209, 1.181, 1.397, and 1.122, respectively. The results of the correlation analysis showed that leader ethical behavior was associated with leader identification (*r* = 0.897, *p* < 0.001), and radical innovation (*r* = 0.726, *p* < 0.001) and promotion focus (*r* = 0.788, *p* < 0.001) have a positive correlation. Additionally, leader identification was positively correlated with radical innovation (*r* = 0.693, *p* < 0.001) and promotion focus (*r* = 0.780, *p* < 0.001). There was also a positive correlation between radical innovation and promotion focus (*r* = 0.783, *p* < 0.001). Table 2 shows the results of the descriptive statistics and correlation analysis.

**Table 2:** The result of descriptive statistics and correlation analysis.

**Variables**	**Mean**	**SD**	**Leader ethical behavior**	**Leader identification**	**Radical innovation**	**Promotion focus**
Leader ethical behavior	5.645	1.209	–
Leader identification	5.703	1.181	0.897^***^	–		
Radical innovation	5.293	1.397	0.726^***^	0.693^***^	–	
Promotion focus	5.698	1.122	0.788^***^	0.780^***^	0.783^***^	–

*** p < 0.001;

** p < 0.01;

* p < 0.05.

To verify possible problems with multicollinearity, a linear regression analysis was performed using SPSS software. The results show that the value of the variance inflation factor (VIF) of leader ethical behavior is 5.675, leader identification is 5.506, and promotion focus is 2.842. Furthermore, when leader identification is used as a media variable, the VIF value is 5.102. As these are all <6, no serious multicollinearity problem is indicated.

### 4.3 Hypotheses tests

SPSS Process Model 4 was used to analyze the mediation effect of leader identification. The results showed that leader ethical behavior has a positive impact on perceptions of leader identification (Estimate = 0.875, *p* < 0.001) and radical innovation (Estimate = 0.618, *p* < 0.001). Additionally, perception of leader identification has a significant impact on radical innovation (Estimate = 0.252, *p* < 0.01). Therefore, Hypotheses 1, 2, and 3 were supported.

Hypothesis 4 proposed that leader identification mediated the relationship between leader ethical behavior and radical innovation. The indirect effect was 0.220. The bootstrapped confidence intervals were Boot LLCI = 0.039 and Boot ULCI = 0.426, as 0 was not included between Boot LLCI and Boot ULCI. These results indicate that the mediation effect of leader identification was significant. Thus, Hypothesis 4 is supported. Table 3 gives the results of the hypotheses tests.

**Table 3:** The results of Process Model 4.

**Path**			**Estimate**	**SE**	***t***	***p***	**LLCI**	**ULCI**
Leader ethical behavior	→	Leader identification	0.875	0.022	38.905	0.000	0.8315	0.9200
Leader ethical behavior	→	Radical innovation	0.618	0.092	6.673	0.000	0.4362	0.8006
Leader identification	→	Radical innovation	0.252	0.094	2.656	0.008	0.0654	0.4386
**Indirect effect**			**Effect**	**Boot SE**	**Boot LLCI**		**Boot ULCI**	
**Indirect effect(s) of X on Y**								
Leader ethical behavior → Leader identification → Radical innovation			0.220	0.099	0.039		0.426	

### 4.4 Descriptive moderating role of promotion focus

This study tested the moderating role of promotion focus on the relationship between leader identification and radical innovation and on the relationship between leader ethical behavior and radical innovation. The moderation model was examined using SPSS PROCESS Macro 3.4.1 Model 1 and was tested using 95% confidence intervals and 5,000 bootstrapping re-samples. Tables 4, 5 provide the results of the analysis for Hypotheses 5 and 6. The conditional effects of the focal predictor at values of the moderator(s) are: −1 SD, mean (M), and +1 SD. Since 0 was not included between Boot LLCI and Boot ULCI at the level of −1 SD (standard deviation), mean level (M), and mean +1 SD (standard deviation) confidence intervals, it was concluded that statistical significance was confirmed.

**Table 4:** The results of Process Model 1.

**Moderator**	**Level**	**Conditional effect**	**Boot SE**	***t***	***P***	**Boot LLCI**	**Boot ULCI**
**Dependent variable: radical innovation**							
Promotion Focus	−1 SD (−1.122)	0.280	0.059	4.718	0.001	0.163	0.396
	M	0.364	0.058	6.287	0.000	0.250	0.478
	+1 SD (1.122)	0.449	0.066	6.856	0.000	0.320	0.578
	**Estimate**	**Boot SE**		***t***	***P***	**Boot LLCI**	**Boot ULCI**
**Interaction: Leader ethical behavior** × **Promotion focus**							
	0.076	0.021		3.638	0.000	0.035	0.116

**Table 5:** The results of Process Model 1.

**Moderator**	**Level**	**Conditional effect**	**Boot SE**	***t***	***P***	**Boot LLCI**	**Boot ULCI**
**Dependent variable: radical innovation**							
Promotion focus	−1 SD (−1.322)	0.205	0.060	3.407	0.001	0.087	0.323
	M	0.292	0.060	4.859	0.000	0.174	0.410
	+1 SD (1.322)	0.379	0.068	5.536	0.000	0.244	0.513
	**Estimate**	**Boot SE**		***t***	***P***	**Boot LLCI**	**Boot ULCI**
**Interaction: leader identification** × **promotion focus**							
	0.078	0.021		3.742	0.000	0.037	0.118

Hypothesis 5 tested the moderating effect of promotion focus on the relationship between leader ethical behavior and radical innovation, with an interaction of 0.076. The bootstrapped confidence intervals (Boot LLCI = 0.035, Boot ULCI = 0.116) did not include 0, supporting the hypothesis. Similarly, Hypothesis 6 tested the moderating effect of promotion focus on the relationship between leader identification and radical innovation, with an interaction of 0.078. The bootstrapped confidence intervals (Boot LLCI = 0.037, Boot ULCI = 0.118) also did not include 0, supporting this hypothesis as well.

**Figure 2:**
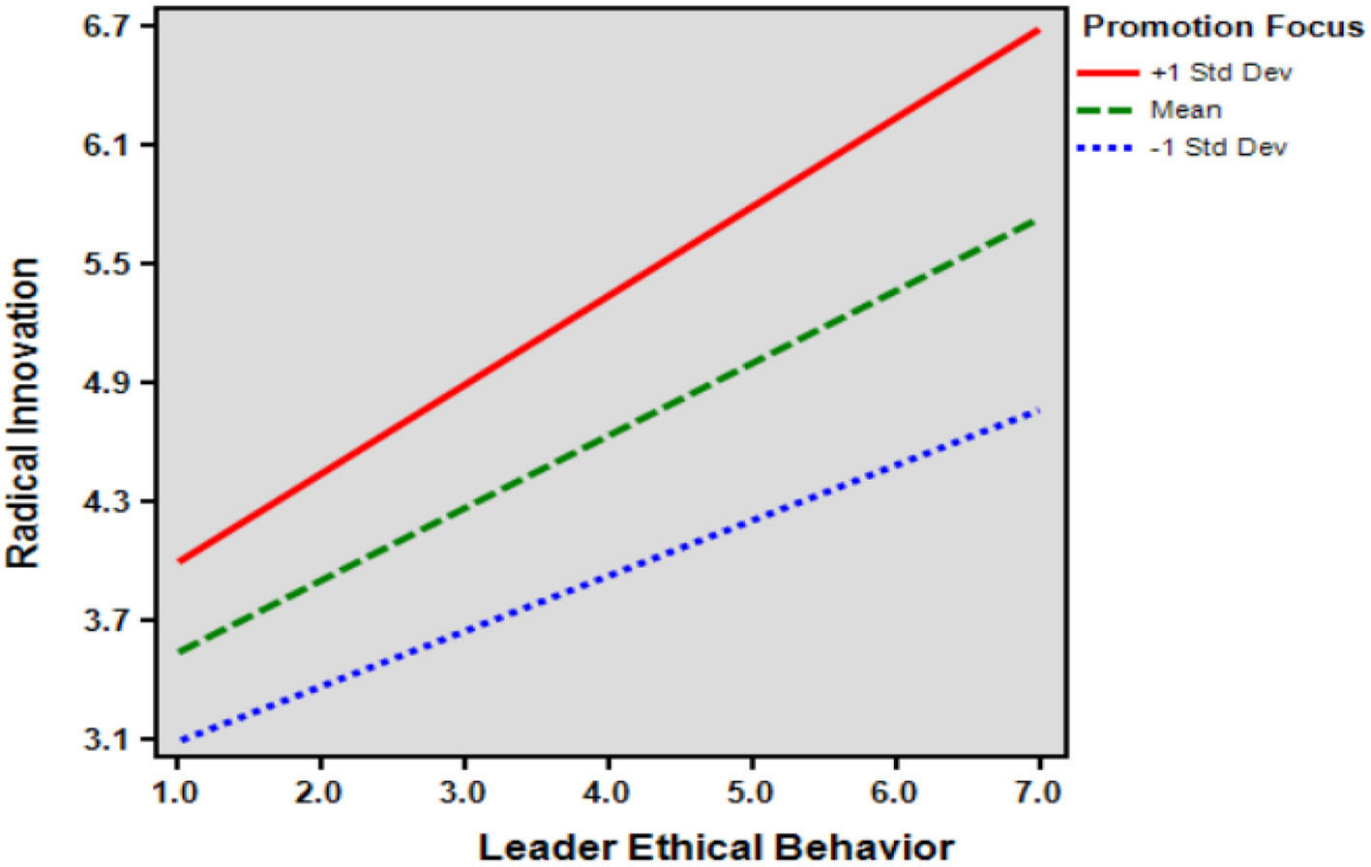
The moderating effect of promotion focus.

**Figure 3:**
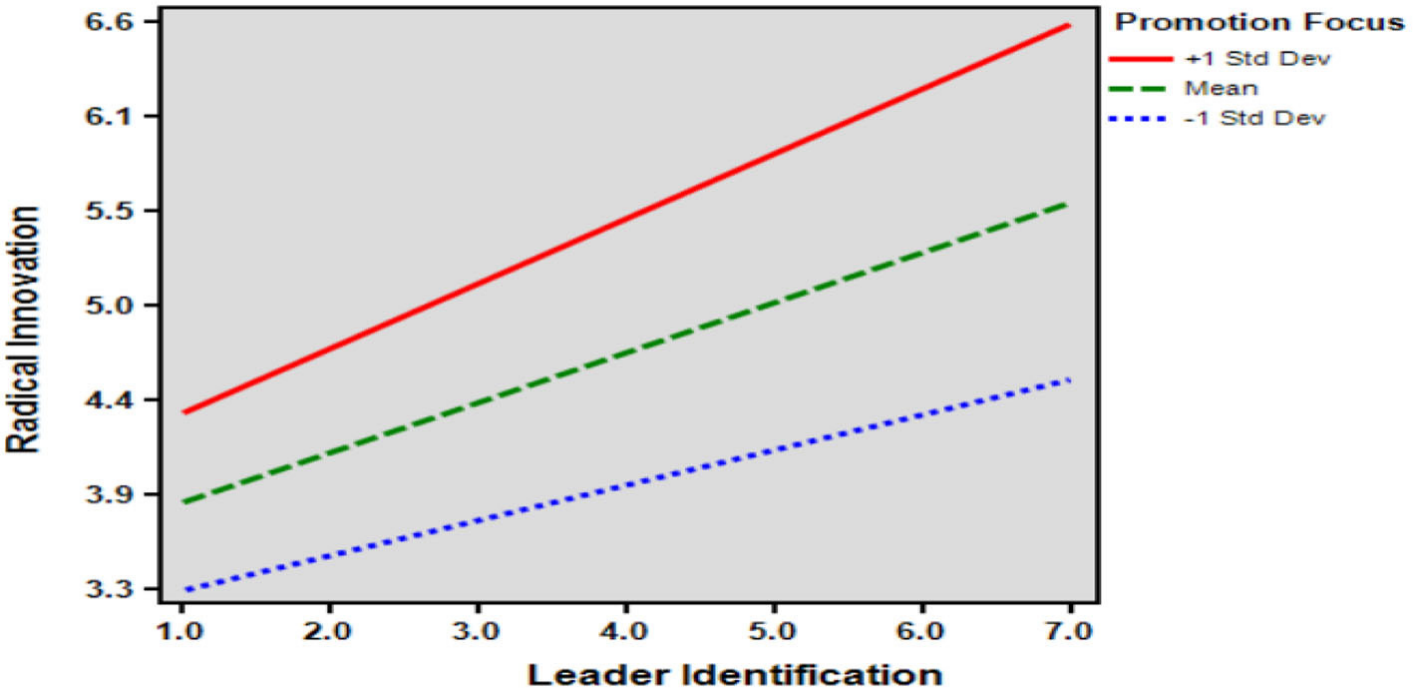
The moderating effect of promotion focus.

As shown in Table 6, the results of the tests for leader ethical behavior (*t* = 0.971; *p* = 0.332 > 0.05), leader identification (*t* = 0.586; *p* = 0.558 > 0.05), promotion focus (*t*=.184; *p* = 0.854 > 0.05), and radical innovation (*t* = 1.510; *p* = 0.132 > 0.05) did not reach the level of significance, which means that the results of males and females' perception of Leader ethical behavior, Leader identification, Promotional focus, and Radical innovation are not significantly different from each other, and it does not affect the results of the questionnaire because of the difference in the number of males or females. The moderation effect diagrams can be referred to Figures 2, 3.

**Table 6:** Independent samples test.

**Variable**	**Equality of variances**	**F**	**Sig**.	***T***	**df**	**Sig. (2-tailed)**	**Mean difference**	**95% Confidence interval (Lower–Upper)**
Leader ethical behavior	Equal variances assumed	0.274	0.601	0.971	369	0.332	0.124	(−0.12763, 0.37681)
	Equal variances not assumed			0.980	324.313	0.328	0.124	(−0.12554, 0.37471)
Leader identification	Equal variances assumed	0.065	0.799	0.586	369	0.558	0.073	(−0.17303, 0.32003)
	Equal variances not assumed			0.590	321.858	0.556	0.073	(−0.17156, 0.31857)
Promotion focus	Equal variances assumed	1.053	0.305	0.184	369	0.854	0.021	(−0.21244, 0.25620)
	Equal variances not assumed			0.183	309.770	0.855	0.021	(−0.21373, 0.25748)
Radical innovation	Equal variances assumed	0.333	0.564	1.510	369	0.132	0.223	(−0.06759, 0.51425)
	Equal variances not assumed			1.517	320.569	0.130	0.223	(−0.06622, 0.51288)

## 5 Discussion

This study's purpose was to explore the mechanisms that trigger employee radical innovation in Chinese SMEs. This study, grounded in the theoretical frameworks of relational identification and social exchange, explores the relationship between leader ethical behavior, leader identification, and employee behavior. It establishes a logical connection between leader ethical behavior and radical innovation through leader identification. The findings suggest that leader ethical behavior fosters positive relationships between leaders and employees, motivating employees to reciprocate through higher levels of radical innovation.

The results also indicate that employees who have a stronger sense of leader identification are more likely to engage in radical innovation as a form of reciprocal behavior when they perceive leader ethical behavior. This study contributes to our understanding of radical innovation by illustrating the role of leader ethical behavior and the mediating effect of leader identification. The implications are valuable for organizations looking to promote radical innovation by emphasizing leader ethical behaviors. In addition, we validate the moderating role of promotion focus. The findings suggest that employee promotion focus moderates not only the relationship between leader ethical behavior and radical innovation but also that between leader identification and radical innovation. This study focuses on validating a model of what triggers employee radical innovation and provides a valuable contribution to the in-depth study of radical innovation. The study has the following theoretical and practical implications.

### 5.1 Theoretical implications

First, the study investigated leader ethical behavior's effect on leader identification and radical innovation. The study's results show that leader ethical behavior positively affects leader identification and radical innovation. Leader ethical behavior helps employees realize that they are in a social exchange relationship with their leaders because they are treated fairly and with care. When employees attribute various positive qualities and behaviors to an ethical leader, such as integrity, selflessness, and commitment to their work, strong leader identification follows (Gu et al., 2015). Therefore, as a result of leader ethical behavior, employees feel their leader's care and support, which will stimulate their trust in their leader. In turn, this strengthens the leader's charisma and increases employees' positive perceptions, stimulating a high level of leader identification. In addition, leaders' care and concern for employees and their fair and ethical treatment will motivate employees to pay more attention to the value of their work. Through this increased attention to value, they will generate and apply new ideas and find new ways to achieve organizational goals. Consequently, innovative attitudes in the organizational environment will increase their creativity, leasing to radical innovation (Shafique et al., 2020). Therefore, leader ethical behavior positively impacts radical innovation.

Second, this study clarifies the strong link between leadership identification and radical innovation and validates leadership identification's effect on radical innovation. The study results show that leader identification has a significantly positive effect on radical innovation. Employees with high leader identification regard their leaders as self-references and role models and actively participate in creative activities by learning and imitating leader behaviors (Wen et al., 2017). This internalization process causes employees to view their leader's goals as their own, consequently motivating them. This motivation drives employees to further pursue and meet their leaders' expectations through positive behaviors. This process can potentially produce radical innovation.

Third, the study verified leader identification's mediating role in the relationship between leader ethical behavior and radical innovation. The results show that leadership identification significantly affects leader ethical behavior and radical innovation and that leader ethical behavior positively affects radical innovation through leader identification. Leader identification helps employees establish a strong emotional connection with their leaders, enhances their emotional attachment to their leaders, and leads them to more strongly identify with their leaders' values and behaviors (Cao and Xue, 2023). Leaders who demonstrate the moral qualities of integrity, honesty, objectivity, and fairness are likely to win employee trust and respect, so that they form a sense of identification with the leader. Employees will actively agree with their leader's ideas and hopes and be willing to work hard to achieve the leader's expectations, thus displaying radical innovation behaviors (Ryou and Kim, 2018). Overall, employees feel leaders' profound leadership charisma due to their display of ethical behaviors, such as high quality and emotional care. Leaders not only earn employee respect and admiration, but also establish a sense of empathy and leader identification. This emotional connection stimulates employees' intrinsic motivation and pushes them to participate in the organization with a positive attitude, generating radical innovation that benefits the organization.

Finally, this study verified the moderating role of promotion focus in the relationship between leader ethical behavior and radical innovation and that of leader identification and radical innovation. The study results indicate that promotion focus has a significantly positive effect not only in moderating the relationship between leader ethical behavior and radical innovation but also in moderating the relationship between leader identification and radical innovation. Employees with promotion focus give organizations an innovation advantage because they are more positive in their approach to the environment, events, and opportunity assessment; this makes it easier to consider new possibilities and generate new ideas when developing innovative products, services, and business models (Li and Shang, 2011). The moderating role of the facilitation focus means that companies can inspire employees to engage in radical innovation by reinforcing leader ethical behavior and enhancing leader identification. Because radical innovation involves high risk and uncertainty, employees need psychological security, mission-driven and innovative motivation to challenge conventional thinking and explore cutting-edge areas. When leaders demonstrate high ethical behaviors such as integrity, fairness, and caring for employee development, it enhances employees' sense of trust and psychological security in the organization and reduces their concerns when innovating. For example, with a leader's support, employees are more bold in coming up with disruptive ideas without undue fear of the consequences of failure. In addition, when employees develop leadership identification, they are more willing to take on high-risk challenges and take the initiative to explore cutting-edge technologies, drive industry change, and bring radical innovations to fruition.

### 5.2 Practical implications

In addition to the theoretical contributions, our theoretical and research findings have the following practical implications. First, leader ethical behavior values collective interests, which contributes to forming an organizational climate of candor, sharing, and inclusion and subsequently reduces organizational conflicts (Cheng et al., 2023). Moreover, by demonstrating ethical behavior to satisfy employees' psychological needs, leaders stimulate good exchange relationships between employees and leaders; employees respond by displaying positive behaviors that benefit the organization (Yan et al., 2022). Therefore, leaders should fully recognize that their own behaviors have a significant impact on employees' attitudes, behaviors, and organizational atmosphere. To cultivate leader ethical behavior that promote radical innovation, firstly, leaders should set an example of ethical decision-making and emphasize the importance of maintaining integrity, transparency, and responsibility in the innovation process. This will help ensure that innovation is not achieved at the expense of ethical standards. Secondly, leaders should create a trusting and open organizational environment that encourages free exchange of ideas, fosters trust among employees, and enables them to feel safe, thus speaking freely and expressing their opinions freely. Finally, organizations can implement training programs on ethical leadership to help leaders at all levels recognize the positive impact of leadership behaviors. These trainings will help leaders recognize the importance of ethical behavior, prompting them to follow ethical standards when making decisions and respect employees' opinions, thereby promoting innovation while encouraging employees to actively participate in the overall development of the organization.

Second, leader identification reinforces employees' positive emotions at work, actively engaging them in their work (Liao et al., 2022). When employees develop leader identification, they internalize the leader's values and goals and consider the leader's focus on the organization's goals as their own, thus actively improving their own work performance. Leader identification is based on mutual respect, trust, and effective communication. Therefore, leaders should actively listen to employees' opinions and feedback, fully respect their viewpoints, and seriously consider employees' suggestions when making decisions. Giving employees the appreciation and recognition they deserve in a timely manner helps them believe that their work and efforts are genuinely recognized and valued. This positive feedback not only shows that their contributions are valued and respected, but also enhances their trust in and satisfaction with their leaders. These positive interactions inspire employees to identify with their leaders, creating a more positive work atmosphere.

Third, Employees with promotion focus pursue desirable goals and achieve their desired states by pursuing success, leading to progress, growth, and fulfillment (Crowe and Higgins, 1997). The moderating role of promotion focus implies that leaders should stimulate the promotion focus of their employees to enhance their motivation to innovate and their willingness to develop professionally. This can be done through incentives, organizational culture shaping, and goal setting to optimize management decisions and drive organizational innovation. Leaders should understand employees' career goals, provide training and growth opportunities, and fairly recognize their achievements and provide incentives to enhance their sense of belonging and commitment to innovation. Under a relaxed innovation climate and performance reward system, employees with a high promotion focus are more willing to explore new opportunities, take risks, and drive change. In addition, encouraging innovative thinking and a spirit of trial and error can help increase job satisfaction and promote long-term organizational growth. Therefore, companies should strengthen the promotion focus in incentive policies, performance appraisals and strategic planning to provide employees with development opportunities and promote win-win situations for both individuals and organizations.

Finally, radical innovation is risky for organizations but can bring them great rewards (Colombo et al., 2017). In China, SMEs tend to be smaller in scale and relatively weaker in financial strength, making them more vulnerable to business risks. To survive and thrive in an increasingly complex and competitive market environment, Chinese SMEs must demonstrate a strong commitment to innovation and focus on developing unique competitive advantages (Chen et al., 2012). This study highlights the crucial role of innovation for Chinese SMEs, aiming to help them better adapt to market dynamics. To achieve this, managers must find ways to encourage their employees' engagement in innovation, especially radical innovation. First, leaders should actively encourage creative thinking. Motivating employees to conceive novel ideas creates an organizational climate that is open and supportive of innovative development. Second, leaders should ensure that they allocate sufficient resources to R&D (research and development) and technological innovation to keep the organization at the forefront of innovation. Finally, leaders should pay attention to their employees' psychological states, listen to their views and difficulties, and promptly address any uneasiness and concerns that may arise during the process of employee radical innovation. Such care can enhance employee confidence and motivation and result in smoother innovation processes.

### 5.3 Limitations and future research

Although this study confirmed the relationship between leader ethical behavior and radical innovation, it has certain limitations, which are detailed below. In addition, we suggest directions for future research.

First, the study respondents all worked in Chinese SMEs. Future research could focus on examining SME employees in different cultural contexts to verify whether the results are similar to those in this study. A comparative study will deepen our understanding of the relationship between leader ethical behavior and radical innovation and provide a more comprehensive perspective of leadership behavior in SMEs.

Second, the only moderating variable considered in this study is promotion focus. In future research, in addition to considering promotion focus, the moderating role of prevention focus should be explored. Applying these two types of focus together will allow in-depth study of the effects of leadership behavior and identification on radical innovation to verify their specific moderating effects in this relationship.

Additionally, as a cross-sectional study, this study only measured a single point in time. The cross-sectional design restricts causal inferences. To improve the study's accuracy and depth, our future research will adopt a longitudinal research design to gain a more comprehensive understanding of time trends and the level of leader ethical behavior's influence on radical innovation by taking measurements at multiple points in time. Adopting this research methodology will enable drawing more reliable conclusions and reveal underlying causal relationships, thereby increasing the credibility of the findings.

Fourthly, this study exhibits a certain imbalance in gender distribution, with a relatively lower proportion of male participants, which may affect the generalizability of the findings. To minimize the potential impact of gender as a dummy variable, future research could adopt a more balanced gender ratio during the data collection phase to enhance the representativeness, robustness, and applicability of the study.

Fifth, although the confirmatory factor analysis (CFA) generally supports the measurement model of this study, the RMSEA value of 0.095 indicates that there is still room for improvement in the model fit. While the overall model remains within an acceptable range, future research could enhance the robustness and generalizability of the study by improving item selection, exploring more optimal measurement models, or validating the model using larger and more diverse samples.

Sixth, this study explored the impact of leader ethical behavior on employees' radical innovation and reached relevant conclusions. However, we must acknowledge the potential omission of certain variables that may influence radical innovation. For example, previous studies have highlighted the significant impact of authentic leadership, quality of leadership, and work-related stress on employee behavior (Fu et al., 2022; Van der Heijden et al., 2017; Mucci et al., 2015). Recognizing these factors not only reveals the limitations of this study but also provides a direction for future research. Therefore, future studies can further investigate the specific effects of authentic leadership, quality of leadership, and work-related stress on employees' radical innovation to validate their roles in driving innovation, thereby enriching and refining the existing theoretical framework.

Finally, this study relies on self-reported measures, there is a potential risk of common method bias (CMB). To improve its accuracy and credibility, future studies may consider a split method in which leaders report on matters related to their employees and employees report on matters related to their leaders. This can reduce the potential impact of common method bias on the study results and enhance the study's scientific and persuasive nature.

## Data Availability

The raw data supporting the conclusions of this article will be made available by the authors, without undue reservation.
